# Etiology, clinical presentation, and outcome of temporomandibular joint luxation in cats: 21 cases (2000–2018)

**DOI:** 10.3389/fvets.2023.1274193

**Published:** 2023-11-03

**Authors:** Jin W. Jung, Ana C. Castejón-González, Darko Stefanovski, Alexander M. Reiter

**Affiliations:** ^1^Tribeca Veterinary Dentistry and Oral Surgery, New York, NY, United States; ^2^Department of Clinical Sciences and Advanced Medicine, School of Veterinary Medicine, University of Pennsylvania, Philadelphia, PA, United States; ^3^Department of Clinical Studies-New Bolton Center, School of Veterinary Medicine, University of Pennsylvania, Philadelphia, PA, United States

**Keywords:** temporomandibular joint, luxation, TMJ, cat, feline, trauma, computed tomography, CT

## Abstract

This study was performed to report etiology, clinical presentation, treatment, and outcome of temporomandibular joint (TMJ) luxation in 21 cats (2000–2018). TMJ luxation was diagnosed by either dental radiography or computed tomography (CT). Causes for TMJ luxation included altercation with a dog (38%), hit-by-car (19%), unknown (19%), fall (19%), and ran into inanimate object (5%). The most common complication was development of permanent malocclusion (24%), followed by reduced vertical mandibular range of motion (5%). Reduction of TMJ luxation was successful in 94.7% of the cases. Reduction of the TMJ luxation was significantly associated with time from injury to treatment. No other significant associations were observed between cause of injury, type of TMJ stabilization, and outcome. TMJ luxation in cats carries an excellent prognosis with early joint reduction and supportive care. Concurrent dental trauma and maxillofacial injuries are common, and all patients with head trauma must be stabilized and evaluated with a thorough examination.

## Introduction

The temporomandibular joint (TMJ) is a synovial condylar joint which enables vertical movement of the mandible for mastication (1). The head of the mandible of the condylar process articulates with the mandibular fossa of the squamous part of the temporal bone. The mandibular fossa contains two bony protuberances: the retroarticular process and the articular eminence. The retroarticular process is a caudoventral extension of the mandibular fossa which cups the head of the mandible of the condylar process, and the articular eminence of the mandibular fossa is an extension at its rostral margin (2). The TMJ is surrounded by an articular capsule, which is further stabilized by the lateral ligament and the lateral pterygoid muscle (1, 2). A recent study showed that the capsule is also reinforced by a caudal ligament in cats (3). A fibrocartilaginous disc divides the intraarticular space in a dorsal and ventral compartment.

TMJ luxation is referred to as a complete dislocation in which the articular surfaces of the joint are separated. A subluxation is a partial or incomplete dislocation (4). The direction of dislocation can be defined as the position of the head of the mandible of the condylar process relative to the mandibular fossa (e.g., rostrodorsal or caudoventral). Luxation of the TMJ is more common in cats than in dogs and often results from traumatic incidents such as altercation with dogs, motor vehicle accidents, and falls from a height (5). Patients with unilateral rostrodorsal TMJ luxation often present with acute inability to fully close the mouth, drooling saliva, and a rostral deviation of the affected mandible and shift to the opposite side of the dislocation. However, TMJ luxation remains a diagnostic challenge among veterinarians, given that other causes of inability to close the mouth may have similar clinical presentations. Other conditions with similar presentation (inability to close the mouth with or without malocclusion) are tooth luxation, maxillofacial trauma with mandibular or maxillary fractures, oral and maxillofacial neoplasia and open- mouth jaw locking (6). Furthermore, general practitioners may lack important diagnostic imaging capability or experience in interpreting TMJ radiography and computed tomography (CT).

Despite an oral examination that can guide us to the diagnosis, only diagnostic imaging provides a definitive diagnosis of TMJ luxation. In addition to a thorough oral examination, proper assessment of the patient’s neurologic status and airway integrity is of paramount importance. Many cases of TMJ luxation are associated with head trauma (7, 8), and therefore animals diagnosed with TMJ luxation often present with concurrent dental trauma and other maxillofacial injuries (9). Concurrent injuries must be detected early on because they can change treatment options, lead to potential complications, and may affect the clinical outcome. After appropriate initial stabilization of a patient and diagnosis of TMJ luxation, closed joint reduction followed by maxillomandibular approximation (MMA) or fixation (MMF) may allow cats to regain normal occlusion and masticatory function. The purpose of this retrospective case series is to describe the clinical presentation, signalment, causes, lesion location, treatment, and outcome of TMJ luxation in 21 client-owned cats.

## Materials and methods

Medical records of cats presented to the Matthew J. Ryan Veterinary Hospital of the University of Pennsylvania (MJR-VHUP) and diagnosed with TMJ luxation from 2000 to 2018 were reviewed. Patients were included in the study if TMJ luxation was diagnosed by radiography or CT. Cats with fracture of the condylar process or mandibular fossa on the same side of TMJ luxation were excluded in the study.

Information about signalment and weight of the patient, cause, diagnosis, and location of the TMJ lesion, assessment of the occlusion, concurrent dental and maxillofacial injury, imaging findings, and treatment, and outcome were retrieved from the medical records and dental charts. Radiographs, CT images, and clinical photographs, when available, were reviewed. Attempts were made to call the owners of all patients for pertinent follow-up information.

TMJ luxation was characterized as bilateral or unilateral, and rostrodorsal or caudoventral. The time from injury to treatment was recorded in days. Treatment was categorized as (a) closed reduction only, (b) closed reduction with tape muzzling, (c) closed reduction with modified labial buttons and sutures, (d) closed reduction with tension-relieving sutures through intravenous fluid tubing at the commissure of the lips, and (e) closed reduction with bis-acrylic composite interarch splinting between maxillary and mandibular canine teeth.

TMJ reduction outcome was considered successful if the head of the mandible of the condylar process was repositioned in the mandibular fossa radiographically. The occurrence of complications (malocclusion, reduced vertical range of motion) at the follow-up visits was also retrieved from the medical records. The evaluation was performed under anesthesia if the patient needed further treatment (e.g., tooth extraction, removal of MMF, etc.).

All analyses were conducted with Stata 17MP, StataCorp, College Station TX. The descriptive analyses included the computations of means, medians, and ranges. Normality testing (Shapiro–Wilk test) was performed to determine if the data was skewed. The categorical variables were reported as frequency counts and percentages. Univariate Firth logistic regression was used to assess the association between the outcome of interest (complication, malocclusion, and others) and the set of independent variables. The independent variables evaluated were cause, time from injury to treatment in days, presence of articular fracture on the opposite side (1 patient) of TMJ luxation, presence of maxillary fractures or non-articular mandibular fractures, and type of treatment. All associations reported as odds ratio (OR) with their respective 95% CI and *p*-values.

## Results

**Figure 1:**
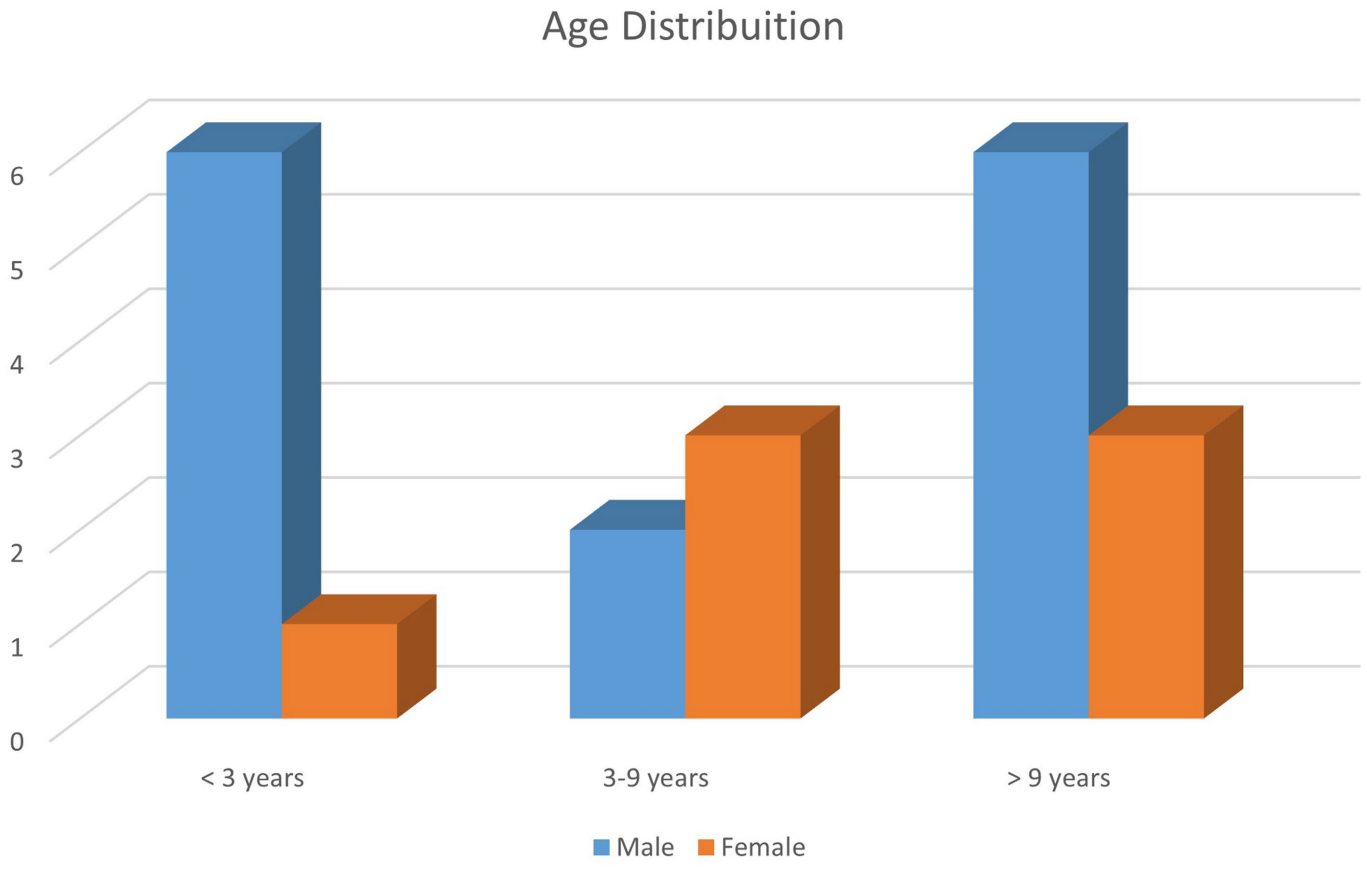
Age distribution of 21 cats with temporomandibular joint (TMJ) luxation.

A total of 21 client-owned cats with 21 TMJ luxations were included in this study. All cats had a known age according to the owner at admission. The median age was 79 months (range, 8–180 months). Five cats (23.8%) were under 18 months of age. Fourteen cats were male, including one intact male, and seven were female (Figure 1). Nineteen cats were domestic breed, and two were purebred cats (one each of British short hair and Bengal breeds). The mean weight of the cats was 5.0 kg (range, 3.0–7.2 kg) at admission and 4.86 kg (range, 3.2–7.2 kg) at follow-up. Three (14.3%) cats received an esophagostomy tube for nutritional support.

**Figure 2:**
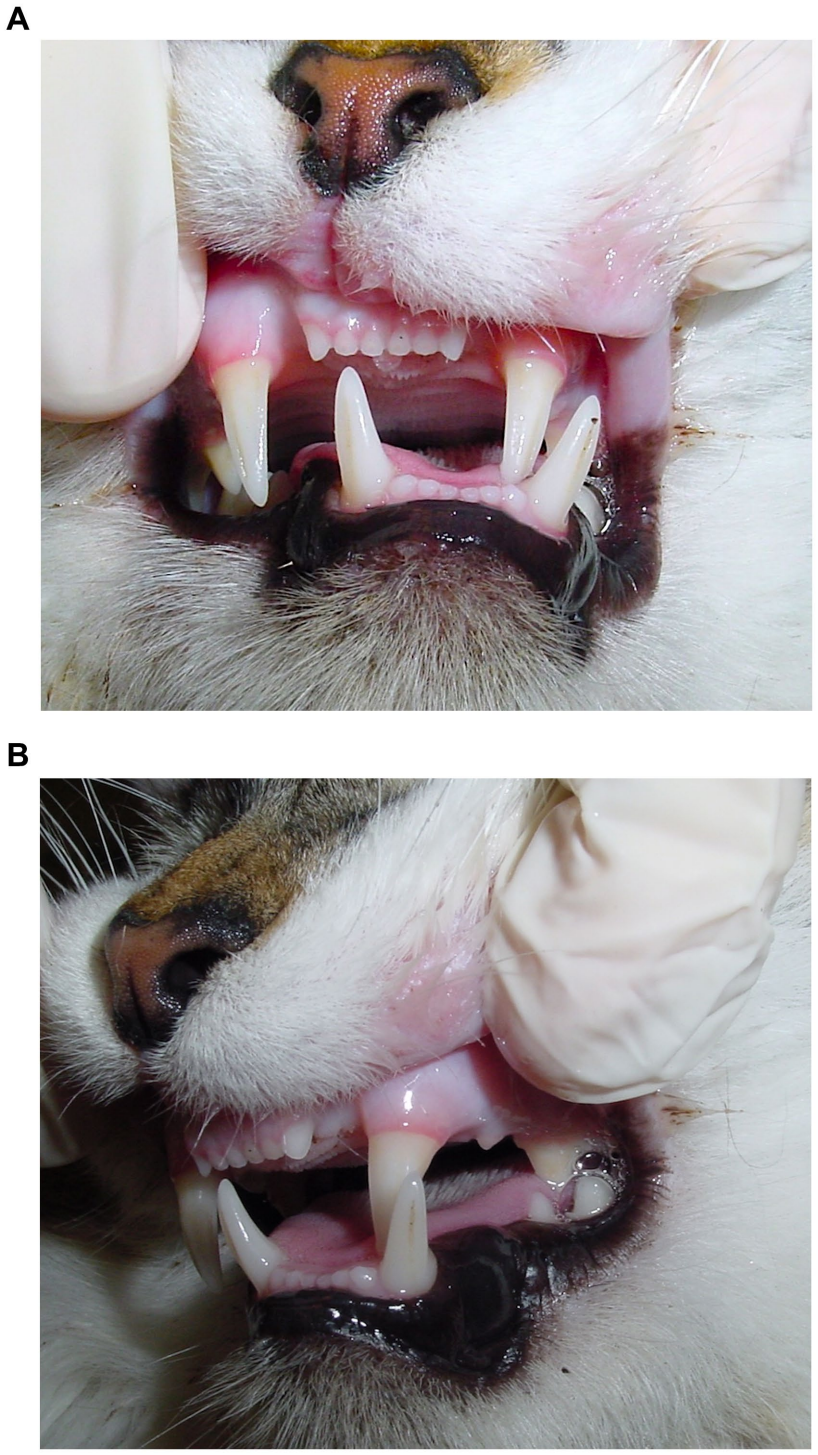
Malocclusion secondary to right temporomandibular joint luxation in an 11-year-old domestic cat. **(A)** Deviation of mandibles towards the opposite side of the luxation. **(B)** Inability to close the mouth due to contact between the left maxillary and mandibular teeth. The left maxillary canine occludes lingual and distal to the left mandibular canine tooth.

**Figure 3:**
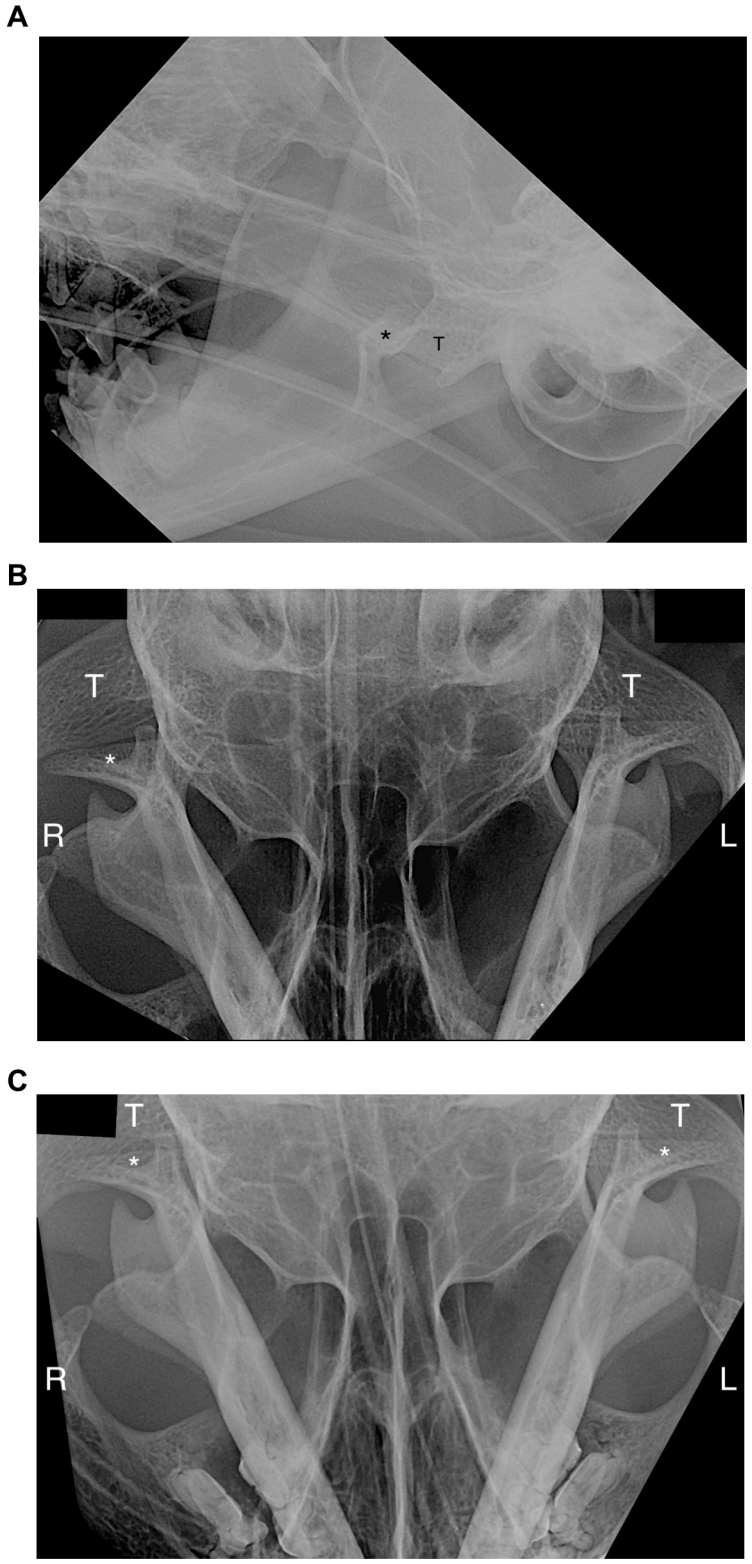
Radiographic view of head of the patient in Figure 1. **(A)** Lateroventral to laterdorsal (LaV-LaDO) view with 10-to-20- degree rotation of the head in the transverse plane around their long axis. The right mandibular condylar process (*) is displaced rostral and dorsal from the mandibular fossa of the temporal bone (T). **(B)** Dorsoventral view of right and left temporomandibular joints (TMJs). The right condylar process (*) is not overlapped with the mandibular fossa (T) and it is displaced (luxated) rostrally. The left TMJ is normal. **(C)** Dorsoventral view after reduction of the right TMJ. Right and left condylar processes (*) are overlapped with mandibular fossa of the temporal bone (T). R, right; L, left.

**Figure 4:**
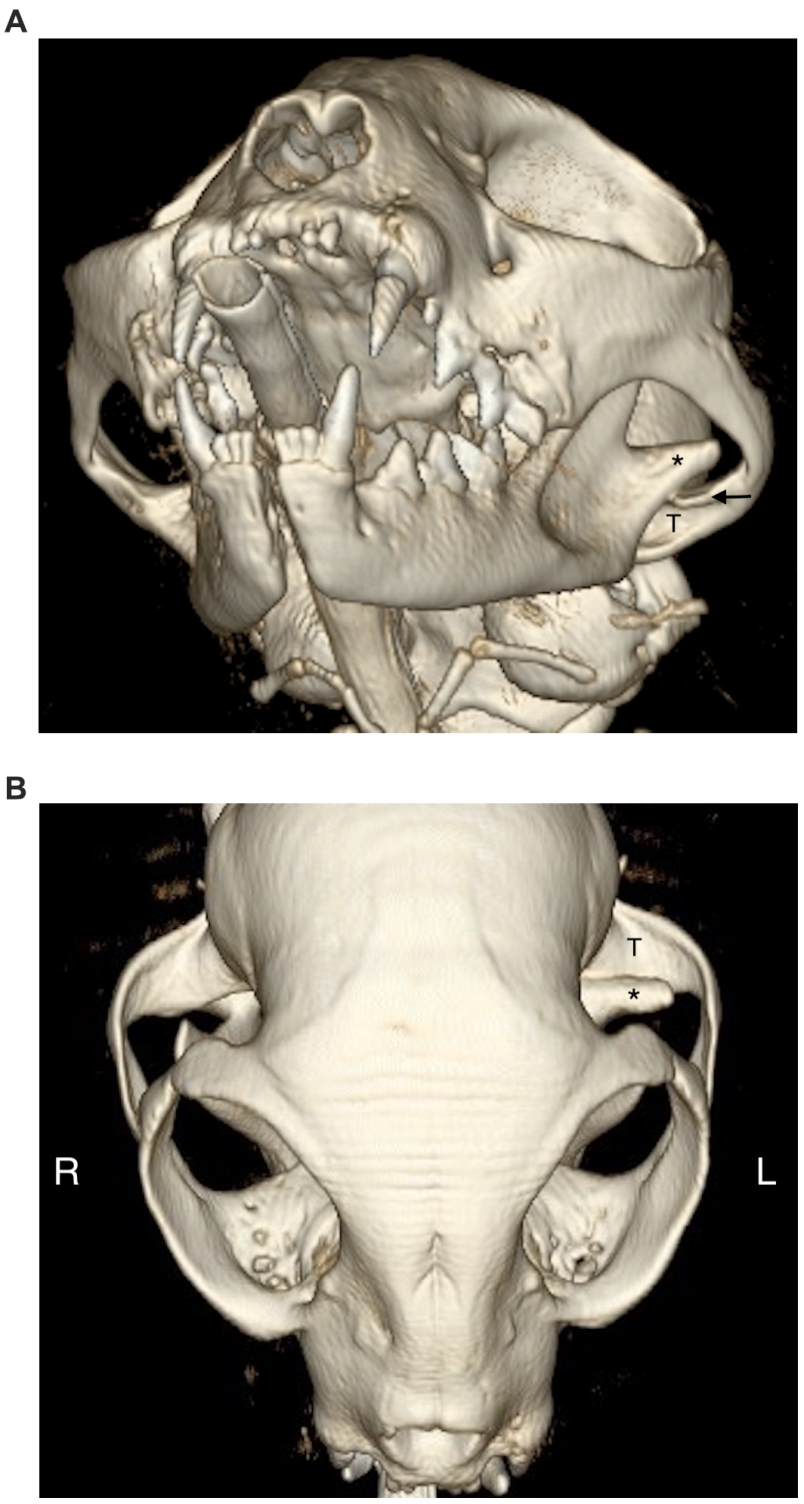
3D rendering of a patient with left TMJ luxation. **(A)** The left condylar process (*) is displaced rostral and dorsally from the mandibular fossa (T). The articular eminence (arrow) limits the mandibular fossa dorsally. **(B)** On the dorsal view the articular eminence is not visible due to the location of the condylar process dorsal to it.

Causes for TMJ luxation included altercation with a dog (*n* = 8; 38.0%), hit-by-car (*n* = 4; 19.0%), fall from a height (*n* = 4; 19.0%), ran into an inanimate object (radiator) (*n* = 1; 5.0%), and unknown trauma (*n* = 4; 19.0%) (Table 1). Ten luxations occurred on the right side and 11 on the left side. Rostrodorsal displacement (*n* = 19; 90.5%) was the most common orientation; this included three cats with rostrodorsal luxation confirmed only with dorsoventral/ventrodorsal (DV/VD) dental radiographs (Figures 2, 3). Caudoventral luxation (*n* = 2; 9.5%) was less common. One of 2 caudoventral luxations was confirmed by CT and neither of them had concurrent articular fracture. All 21 cats had dental radiographs obtained, and six of them also had a CT scan performed (Figure 4).

**Table 1:** Causes of temporomandibular joint (TMJ) luxation in 21 cats and presence of concurrent dental and maxillofacial injuries in each group.

	Altercation with a dog	Hit-by-car	Fallen from a height	Ran into radiator	Unknown
Cats	38% (**8**/21)	19% (**4**/21)	19% (**4**/21)	5% (**1**/21)	19% (**4**/21)
Concurrent dental and maxillofacial injuries for each cause of TMJ luxation	100% (8/**8)**	100% (4/**4**)	100% (4/**4**)	100% (1/**1**)	50% (2/**4**)

Nineteen (90.5%) cats had concurrent dental, oral, and maxillofacial injuries in addition to TMJ luxation. These included mandibular symphyseal separation (*n* = 9; 42.9%), tooth fracture (*n* = 9; 42.9%), lip/oral mucosal bruising or laceration (*n* = 10; 47.6%), maxillary fracture (*n* = 4; 19.0%), ocular and orbital injury (*n* = 4; 19.0%), tongue injury (*n* = 3; 14.3%), mandibular fracture (*n* = 3; 14.3%), other intra-articular TMJ injury such as condylar process fracture on the opposite side of TMJ luxation (*n* = 1; 4.8%), and lip avulsion (*n* = 1; 4.8%). All cats except 2 from unknown cause of TMJ luxation group had concurrent injuries (Table 1). Symphyseal separation was noted in five (55.6%) of 9 cats in the altercation with a dog group. Maxillary fracture was noted in two (50.0%) of four cats in the hit-by-car group. Four (19.0%) of 21 cats had sustained clinically significant ocular injuries ipsilateral to the TMJ luxation, ranging from conjunctival hyperemia to rupture of the globe, and one cat had sustained bilateral ocular injuries. In addition to the obvious ocular injuries, five cats were shown to have reduction of retropulsion of the eye ipsilateral to the TMJ luxation. One cat had increased retropulsion of the eye ipsilateral to the TMJ luxation due to fracture of orbital bones.

The average time from recognition of injury to treatment was 4 days, and the median was 0.5 days (range, 0–60 days). Sixteen (76.2%) cats were brought to the hospital less than 4 days from recognition of injury; one (6.3%) of them developed permanent malocclusion after treatment, 14 (87.5%) of them recovered without any further complications and were reported to have continued good quality of life, and one (6.3%) of them was later euthanized due to unrelated health issues. Four (19.0%) of the 21 cats were brought to the hospital on 4 days or later after recognition of injury, and all (100%) of them had permanent malocclusion after reduction with clear signs of abnormal tooth to tooth or soft tissue contact; two were treated with selective extraction, one was treated with selective extraction, odontoplasty and vital pulp therapy, and one did not return for further treatment. There was no information on the records about when the injury occurred in one cat.

**Figure 5:**
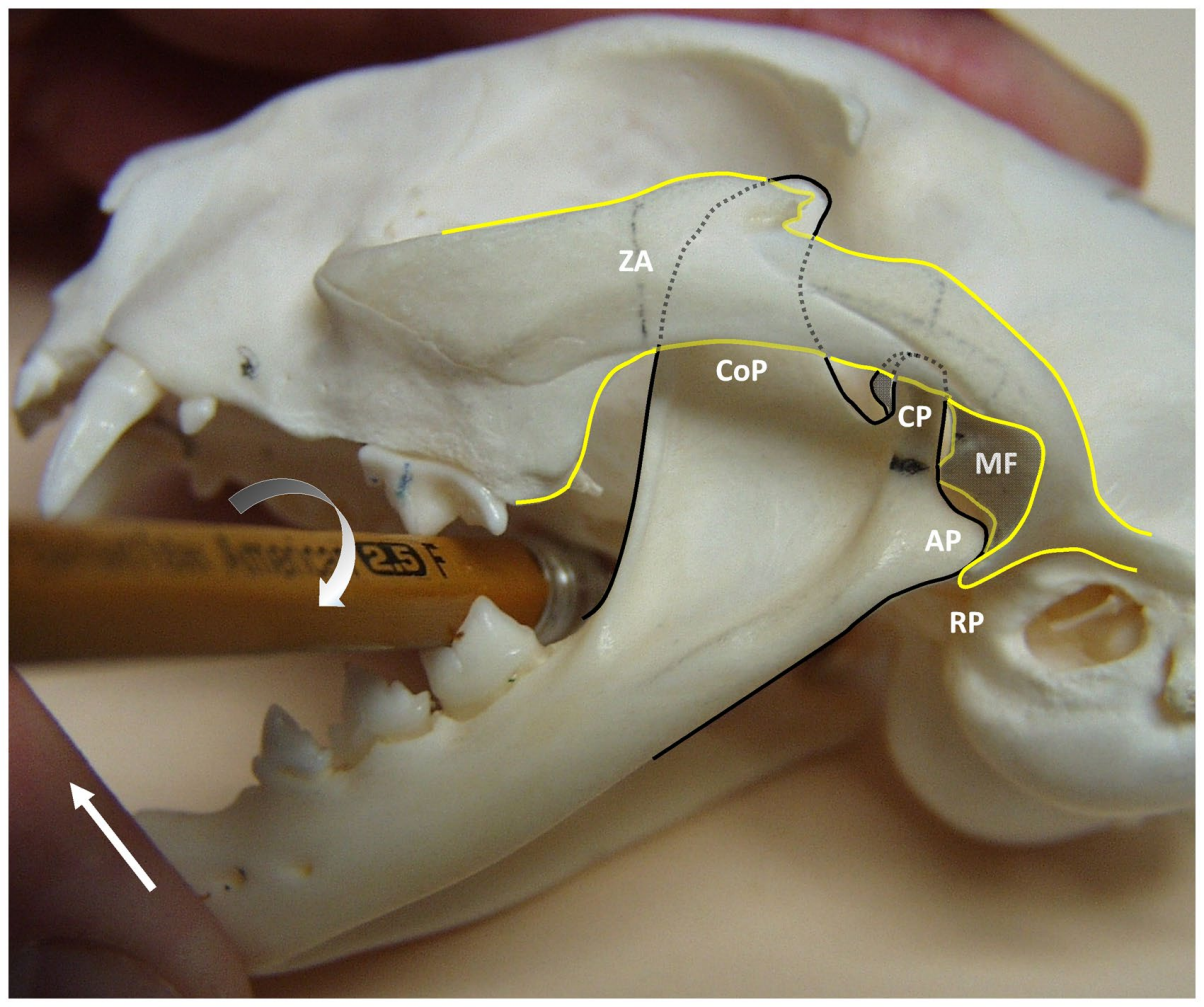
Demonstrating the use of a hexagonal pencil for manual reduction of left-sided rostrodorsal TMJ luxation on a cat skull. The pencil is positioned between the left carnassial teeth (maxillary fourth premolar and mandibular first molar) with one hand and carefully rotated in a clockwise direction (half-circle arrow) while the other hand closes the mouth by manipulating the lower jaw against the upper jaw (arrow). This maneuver moves the left mandible in a rostroventral direction, allowing the head of the mandible of the condylar process (CP) to pop back into the mandibular fossa (MF). ZA, zygomatic arch; CoP, coronoid process; CP, condylar process; MF, mandibular fossa; AP, angular process; RP, retroarticular process.

Closed joint reduction with a hexagonal wooden pencil was attempted in 19 (90.5%) of the 21 cats with TMJ luxation (Figures 3, 5). The TMJ luxation was successfully reduced in 18 (out of 19) (94.7%) of the cats, in which reduction was attempted. The one cat, whose reduction was not successful, acquired TMJ luxation after hit-by-car trauma 60 days prior to presentation. One other cat had multiple mandibular fractures with other concurrent head injuries including symphyseal separation, and closed joint reduction was not attempted. Another cat did not have information in the medical records on whether or not reduction was performed. These three cats had evidence of malocclusion on the initial visit and upon follow-up.

**Figure 6:**
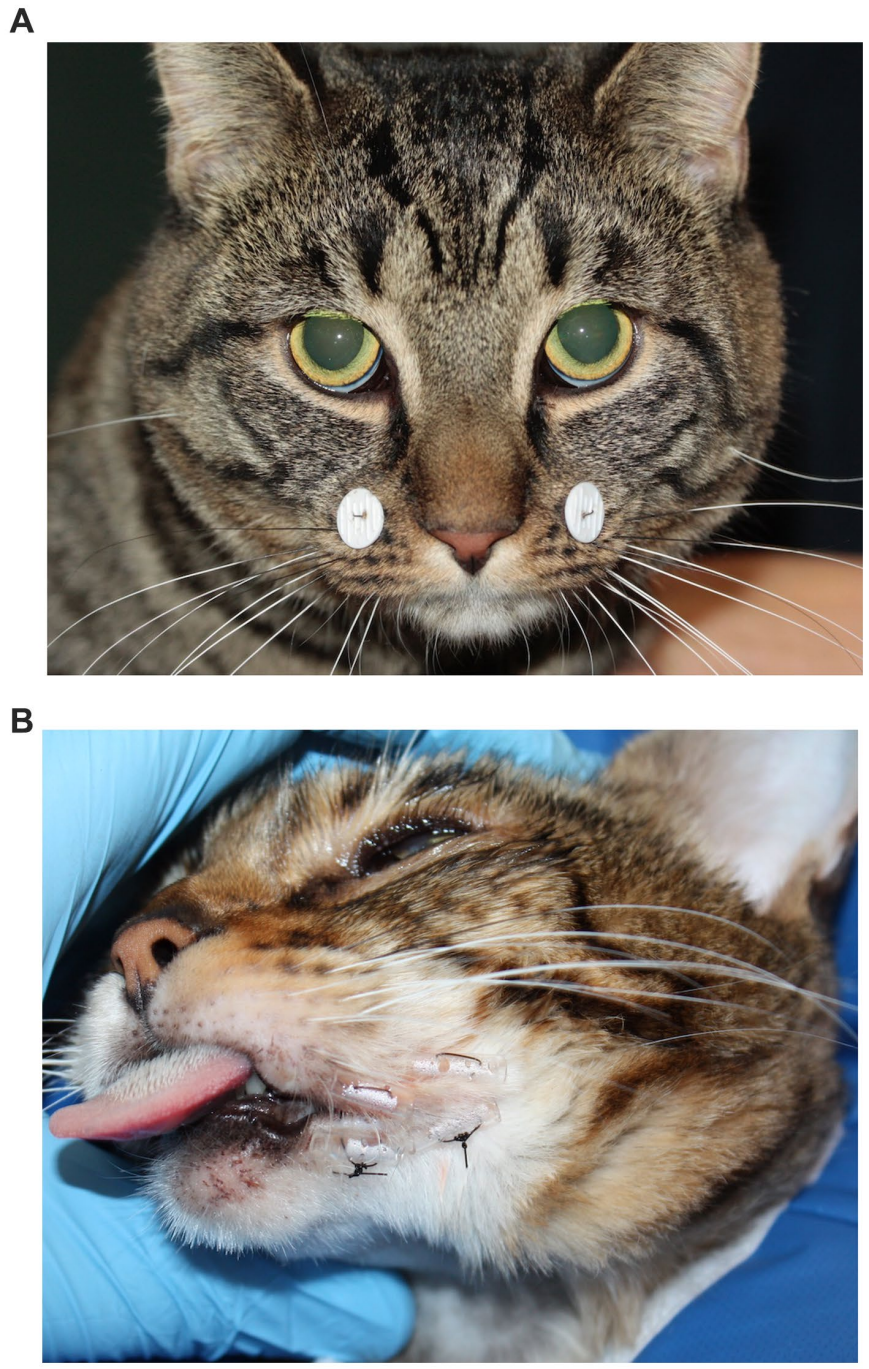
Stabilization methods. **(A)** Modified Labial buttons and sutures technique. **(B)** Temporary commissurorraphy with fluid line tubing and sutures. Both techniques limit the range of mouth opening.

Following successful closed joint reduction, 14 (77.8%) of 18 cats had further stabilization of the TMJ, including tape muzzling (*n* = 7 cats; 38.9%), modified labial buttons and sutures (*n* = 5; 27.8%), tension-relieving sutures through intravenous fluid tubing at the commissure of the lips (*n* = 1; 5.6%), and bis-acrylic composite interarch splinting between maxillary and mandibular canine teeth (*n* = 1; 5.6%) (Figure 6). Four (22.2%) cats after successful joint reduction did not have further stabilization. The mean duration the stabilization stayed in place on these cats was 13 days (range, 0–30 days). The tape muzzles stayed on for 16 days on average (range, 11–21 days). Four cats that had been discharged with tape muzzles did not return for follow-up; when they were followed-up via phone call, the owners did not remember the duration of muzzling and did not report any complication. The labial buttons and sutures stayed on for 14 days on average (range, 0–30 days), and all cats returned for their removal. The interarch splint for one cat stayed on for 4 days.

All 21 cats were available for follow-up (range, 10 days to 84 months). One cat was euthanized after treatment due to unrelated health issues. The most common complication in the 21 patients was malocclusion (*n* = 5 cats; 23.8%) followed by reduced range of vertical mandibular motion (*n* = 1; 4.8%).

Reduction outcome only was significantly associated with time from trauma to treatment (*p* = 0.045). For each day that the treatment was delayed, the likelihood of successful reduction decreased by 6% (OR/day: 0.94, 95% CI: 0.36–0.97). None of the other independent variables evaluated, including cause, presence of TMJ articular fracture on the opposite side of TMJ luxation, presence of maxillary fractures or non-articular mandibular fractures, and type of treatment, were significantly associated with any of the outcomes studied (reduction outcome, complications, malocclusion, and reduced range of vertical mandibular motion).

## Discussion

TMJ luxation in cats commonly occurs due to maxillofacial trauma such as altercation with a dog, motor vehicle accident, or fall from a height (5). It has previously been reported that the majority of traumatic TMJ injuries including luxation were the result of falling from a height and can be accompanied by concurrent injuries (10, 11). In the present study, however, altercation with a dog was the most common cause (38.0%), probably because affected cats were client-owned and mostly indoors. Younger male cats were overrepresented (28.6%, <36 months old) which may be explained by their inquisitive and/or aggressive nature, putting them at a higher risk.

Cats with rostro-dorsal TMJ luxation present with rostral deviation of the mandibles away from the side of lesion, causing an inability to fully close the mouth and drooling saliva due to difficulty in swallowing. These clinical signs and acute anorexia were easily perceived by the owners, and most of the injured cats were brought in to receive medical attention in a timely manner. Time from injury to treatment was significantly associated with successful joint reduction (the condylar process of the mandible was repositioned in the mandibular fossa).

If the luxation is not reduced in a timely manner, inflammatory and fibrotic changes may occur in the joint capsule and articular disc that could interfere with the success of joint reduction and overall TMJ health. In addition, long-standing intra-articular hemorrhage can result in cartilage degeneration and osteoarthritis (12). Statistical analysis showed that chances of success in joint reduction goes down by 6% per 1 day without treatment in the statistical model. However, this result is withdrawn from only one unsuccessful joint reduction case in which the joint reduction was attempted 2 months after the injury. Therefore, a strong conclusion cannot be made from this study regarding how early the TMJ should be reduced to achieve good success. Further studies with more cases are necessary to provide comprehensive validation to this prediction.

What makes a diagnosis of TMJ luxation challenging for veterinarians is that there are multiple other conditions that could result in an inability to close the mouth and may present with similar clinical signs. They may even occur with some TMJ dislocation as shown in the present study. In TMJ luxation, the deviation of the mandibles will depend on the side and displacement orientation of the luxation. Typically, the mandibles will deviate away from the side of lesion with rostrodorsal TMJ luxation. Contrary to that, unilateral mid- and caudal body mandibular fracture may result in deviation of the mandibles towards the side of lesion.

Displacement of the head of the mandible of the condylar process in a caudal direction is limited by the retroarticular process. Caudoventral TMJ luxation is usually present with concurrent fracture of the retroarticular process of the mandibular fossa (10). Caudoventral luxation was diagnosed in 2 cats without evidence of other TMJ injuries in the present study. One of the cases had only dental radiographs, therefore, small fractures or dysplasia of the TMJ may have been missed. The second case, diagnosed by CT, did not have any other injuries or dysplasia. In caudal TMJ luxation, the mandibles will shift towards the ipsilateral side of the injury. Thus, oral examination alone may not be diagnostic in the presence of multiple maxillofacial injuries, and severity and displacement of fracture fragments can interfere with interpretation of presenting clinical findings. Diagnostic imaging is always necessary to obtain a definitive diagnosis.

TMJ luxation can occur as an isolated injury or be associated with concurrent dental and maxillofacial injuries. The rather wide angle between the mandibles in cats (measured from the mandibular symphysis to both TMJs) allows unilateral rostrodorsal TMJ luxation without mandibular fracture or symphyseal separation (13), but dislocation may occur more easily with either symphyseal laxity or mandibular fracture as shown in this study due to increased rotational movement of the mandible and lateral flaring of coronoid process (14). It is also important to detect tooth fractures during a conscious oral examination. Several cats in the present study had concurrent dental injuries, including uncomplicated crown fractures and complicated crown and crown-root fractures. Tooth extraction was the most common treatment performed either at the time of joint reduction or during follow-up visits.

The presence of canine teeth can play an important role in selecting the most appropriate stabilization technique to maintain normal occlusion following joint reduction. Canine teeth work as a natural interlock, so any structural damage that hinders this ability of interlocking may increase the chance of malocclusion. Therefore, tooth fractures should be addressed once all critical injuries have been treated (15). However, complicated crown or crown-root fractures of teeth other than canine teeth should be addressed at the time of TMJ reduction if possible. Potential complications such as anorexia from pain and inflammation/infection can arise from pulp exposure, and these may necessitate placement of a feeding tube. In the present study, three out of 6 (50.0%) cats with complicated crown fracture of canine teeth were later observed to have malocclusion.

A recent study showed ophthalmic injury was the most common concurrent injury among cats that had skull fractures (16). One fifth of the cats in the present study had eye injuries ranging from conjunctival hyperemia to rupture of the globe, suggesting that all cats with TMJ luxation from head trauma should have a thorough ocular examination performed in addition to a neurologic examination. The retropulsion examination should always be integrated into a thorough physical examination for all head trauma cases, especially when TMJ injury is suspected. With rostrodorsal TMJ luxation, the coronoid process on the affected side will be positioned more rostrally and thus impinge on the contents of the retrobulbar space, resulting in reduced globe retropulsion ipsilateral to the TMJ lesion and a pain response during mouth opening.

Head radiography, dental radiography, and CT are the common imaging modalities for diagnosis of TMJ luxation. Cone-beam computed tomography (CBCT) is becoming more available in the field of veterinary dentistry and oral surgery (17). The small size and compressed nature of the cat’s skull make it more difficult to accurately identify anatomic features without overlap (18, 19). Recent study showed that there are significant anatomic variations in TMJ between different skull conformations of cats which also make interpreting TMJ difficult (20). Imaging the TMJ can be a challenge, and unsafe movement of severely injured patients could result in exacerbation of injuries (18). Initially, head radiography can be a helpful tool for evaluating the mandibular body and occlusion (18), and it has been shown to be useful for evaluating the TMJ when employing adequate technique (18). A standard radiographic series of the TMJ includes dorsoventral (DV) or ventrodorsal (VD), laterolateral, and opposite lateral oblique views (18). The oblique views require specific angulation of the head to best visualize the TMJ of interest.

Recently, it has been demonstrated that to better evaluate the TMJ anatomy with radiography, the recommended radiographic view is in a lateroventral to laterodorsal (LaV-LaDO) direction with 10-to-20-degree rotation of the head in the transverse plane around their long axis (21). When evaluating for TMJ luxation on DV or VD views, the key anatomical structure to identify is the retroarticular process and evaluate for any gap rostrally between the retroarticular process and head of the mandible of the condylar process.

Due to aforementioned challenges and concurrent maxillofacial injuries, obtaining CT or CBCT images are considered gold-standard and preferred to head and dental radiography to fully characterize the TMJ injury and other maxillofacial trauma (18, 19). It helps to avoid missing injuries such as maxillary and intraarticular or extraarticular fractures, degenerative changes and TMJ dysplasia (18, 19). In recent studies evaluating the diagnostic yield of different imaging modalities, CT and CBCT were found to be superior for the diagnosis of most maxillofacial injuries, including fracture of the TMJ (17, 18). This clinically important finding has implication for maxillofacial trauma management and surgical planning (17, 22). For surgical planning, CT and CBCT provide excellent bone detail, spatial orientation, and modern post processing algorithms, which allow three-dimensional, sagittal and dorsal reconstructions without superimposition of other structures (18, 20, 22, 23). The present study includes medical records from patients seen for a period of 18 years. Many of these cases did not have a CT performed because it was not readily available. We cannot rule out in those patients which did not have a CT or CBCT the presence of intraarticular fractures of the mandibular fossa or the condylar process or if there were degenerative changes or dysplasia that could have contributed in some degree to the complications (Table 2).

**Table 2:** Summary of concurrent maxillofacial injuries in each patient diagnosed by dental radiographs and CT.

Imaging modality	Patient no.							
		SS	Lip avulsion	Maxillary fracture	Mandibular fracture	TMJ articular fracture	Tooth fracture	Ocular injury
CT and dental radiographs	1	✓					✓	
	4	✓	✓		✓			
	5			✓				✓
	6	✓					✓	
	15	✓						
	17							✓
Dental radiographs	2							
	3							
	7							
	8					✓		
	9							
	10						✓	
	11							
	12	✓			✓		✓	
	13						✓	✓
	14							
	16	✓					✓	
	18	✓		✓			✓	
	19			✓			✓	✓
	20	✓			✓			
	21	✓		✓			✓	

SS, symphyseal separation. TMJ articular fracture (on opposite side of TMJ luxation).

In the present study, joint reduction was highly successful when performed soon after the injury has occurred; however, the risk of unsuccessful joint reduction increases when joint reduction is delayed. Rostrodorsal TMJ luxation can be reduced using a fulcrum such as a hexagonal soft-wooden pencil as previously described (24) (Figure 5). In cases of chronic TMJ luxation with significant fibrosis, open joint reduction, and stabilization by suture imbrication of the joint capsule may be necessary; however, its efficacy in veterinary medicine is questionable without evidence obtained from clinical trials (14).

After joint reduction, restriction of wide mouth opening (to prevent luxation recurrence) and good pain control usually is sufficient for successful recovery. Commonly utilized techniques for stabilization of the TMJ include MMA (muzzling, modified labial buttons and sutures, elastic chain between maxillary and mandibular teeth) or MMF (interarch splinting with bis-acryl composite connecting the maxillary and mandibular canine teeth) (25). MMA is preferred over MMF to allow for some vertical mandibular range of motion, thus reducing the risk of TMJ ankylosis. The goal is to provide stabilization while periarticular tissue fibrosis occurs, allowing the cat to regain normal occlusion and function. The results show that the type of treatment (including the stabilization method) did not affect the complication rate. This may be related to the lack of statistical power of the sample due to small numbers of patients with complications and too many different treatment groups. Another possibility is that the specific anatomy of the TMJ of the cat including concave mandibular fossa with a prominent retroarticular process with the articular head of the condylar process being deeply seated in the mandibular fossa stabilizes the joint once it is reduced with no further trauma during the recovery (3, 23). None of the cases had recurrence of the TMJ luxation during the follow-up. Further studies are required to evaluate the need of stabilization following reduction of the TMJ luxation as this retrospective study cannot determine which method is the most effective or even stabilization is necessary.

The most commonly used stabilization method in the present study was tape muzzling, which is easy to make and readily available. One cat did not tolerate the muzzle, and it had to be removed. Tape muzzles should ideally be replaced every other day to avoid dermatitis from a soiled and wet muzzle. The modified labial button and suture technique was the second most commonly used TMJ stabilization. It may still cause focal dermatitis around the buttons and tracts where the sutures have penetrated through. However, owners do not have to worry about having to change the device in non-compliant patients.

MMA devices may provide less rigid stabilization than MMF methods, but their advantage is that they can be removed by the owners of cats at risk of vomiting, regurgitation, or excessive sublingual and pharyngeal swelling. Those conditions could lead to upper airway obstruction and aspiration pneumonia. MMA devices are especially preferred over MMF methods in immature patients, as they are less likely to interfere with normal development and growth (26). Furthermore, MMA devices are recommended in cases (such as TMJ fracture) where some degree of vertical mandibular motion is desired during recovery to avoid TMJ ankylosis.

Interarch splinting between maxillary and mandibular canine or other teeth may not be the most ideal treatment option for general practitioners due to the necessary dental equipment, materials, and expertise required. Since the mouth is kept slightly open with this rigid MMF technique to allow for water and food intake, cats often experience difficulty swallowing, which could result in acid–base disturbances due to drooling of saliva (27). With any of the MMA and MMF techniques described above, intraoperative anti-emetic medication as well as feeding tube placement and take-home anti-emetic medication should be considered.

Adequate nutritional intake is crucial for the overall well-being of the patient during recovery. An esophagostomy tube was most commonly used for enteral nutritional support in the present study, which is minimally invasive to place, allows feeding immediately after recovery from anesthesia, and is removed easily once nutritional support is no longer needed (28, 29). Esophagostomy tubes have few major complications and are well-tolerated (28, 29). The most common minor complication is inflammation around the stoma, with peristomal abscessation occurring infrequently (29). Nasoesophageal tubes can be used for short-term nutritional support for anorectic hospitalized patients, but they are contraindicated in patients with maxillary fractures (28). None of the cats in the present study experienced postoperative episodes of anorexia, necessitating additional sedation or anesthesia for feeding tube placement during recovery. However, placement of a feeding tube should be considered during the initial surgery for some patients based on current body weight, severity of injury, higher nutritional requirements, and concurrent systemic disease. Patients with less-than-optimal fracture stabilization would also benefit from enteral nutritional support while minimizing movement of fracture fragments.

TMJ luxation in cats usually carries a good to excellent prognosis with a low rate of complications after immediate joint reduction followed by joint stabilization. Complications noted in cats of the present study were malocclusion and decreased mouth opening.

The most common postoperative complication in the present study was malocclusion, which could result in difficulty eating and drinking, dental attrition and pulp exposure, oral ulceration, and TMJ osteoarthritis (30). In a previously reported case of a cat with open-mouth jaw locking, abnormal contact between maxillary and mandibular canine teeth was thought to be the cause of subsequent levering forces and rotational movement of the mandibular body which might have resulted in increased mandibular symphyseal mobility and TMJ laxity (14). Malocclusion has been associated with concurrent maxillary and TMJ fractures because of the altered anatomy of the dental arches (16, 31). In the present study, there were no significant associations between complications, malocclusion or reduced vertical mandibular range of motion, and the presence of maxillary fractures or articular and non-articular mandibular fractures. Considering that all but one of the cases that have complications sustained mandibular or maxillary fractures, it is possible that those injuries were responsible for the malocclusion or decreased range of motion instead of the TMJ luxation. Also, other non-diagnosed injuries of the TMJ that are separate from luxation, may have contributed to these findings. This emphasizes the necessity of advanced imaging in patients with suspected TMJ luxation.

Postoperative management may include a soft diet for 10 to 14 days, activity restriction, analgesic medications, and – if necessary – an Elizabethan collar, with detailed instruction on how to remove a tape muzzle or sutures and buttons in the case of difficulty breathing, vomiting, or regurgitation. Restriction of wide mouth opening for 2 weeks while healing occurs may reduce the risk of postoperative recurrence of TMJ luxation. If TMJ fractures were present, MMA and MMF devices should remain in place for 4–8 weeks (32).

Condylectomy is recommended for unstable and chronic TMJ luxation, in particular when persistent oral discomfort and joint pain are noted. The contralateral TMJ should be closely monitored in patients after unilateral condylectomy due to the altered function with the potential for degenerative changes to occur (33). A 2 week (or sooner) recheck examination is strongly advised. Owners should be instructed to monitor for early signs of ankylosis such as reduced range of vertical mandibular motion, especially during yawning, eating, and drinking (22) if the patient was discharged without MMA or MMF. The extent of mouth opening may gradually improve once normal activity is resumed following removal of MMA and MMF devices.

The present study has some limitations and further consideration related to its retrospective nature. The medical records may not have been complete, and the follow-up visits were not standardized. CT was not available for each patient and therefore, intra-articular fractures may have been missed and none of the cases were further evaluated with CT at follow up to look for degenerative changes of the TMJ. Stabilization methods after joint reduction were not standardized and this could have led to statistical errors. Follow-up generally was too short to determine long-term complications (severity of decreased mouth opening, presence of chronic pain, and occurrence of degenerative changes of the TMJ). Information about slight malocclusion and minor discomfort may not have been recognized or recorded in some cats; therefore, short- and long-term complications may be underestimated. Lastly, the prediction of likelihood of successful reduction by time from injury to trauma from this study must be carefully considered for its clinical application and any other implication as it is based on a small sample.

TMJ luxation in cats can occur after altercation with dogs and hit-by-car trauma. The prognosis is excellent with early joint reduction and supportive care in the absence of other TMJ injury. Concurrent dental and maxillofacial injury is common, and all cats with head trauma must initially receive cardiovascular and respiratory stabilization followed by a thorough physical examination including oral, ocular, and neurologic assessment. Once the patient is stable for general anesthesia, imaging (ideally CT or CBCT) should be performed to obtain a diagnosis and start the most appropriate treatment.

## Data availability statement

The raw data supporting the conclusions of this article will be made available by the authors, without undue reservation.

## Ethics statement

Ethical approval was not required for the studies involving animals in accordance with the local legislation and institutional requirements because ethical approval was not required because it was a retrospective study. Written informed consent was obtained from the owners for the participation of their animals in this study.

## Author contributions

JJ: Writing – original draft, Writing – review & editing. AC-G: Data curation, Supervision, Writing – review & editing. DS: Formal analysis, Writing – review & editing. AR: Data curation, Supervision, Writing – review & editing.
